# Serum prealbumin at admission is associated with post-stroke depression at 3 months in patients with spontaneous intracerebral hemorrhage

**DOI:** 10.3389/fpsyt.2026.1698928

**Published:** 2026-09-10

**Authors:** Hongtao Luo, Qinglian Luo, Xiaoqin Shi, Jiaying Xie, Ruiping Lei, Jie Zhou

**Affiliations:** 1 Department of Neurosurgery, The Affiliated Hospital of Southwest Medical University, Luzhou, Sichuan, China; 2 Department of Neurosurgery, The First People’s Hospital of Yibin, Yibin, Sichuan, China; 3 Department of Ophthalmology, The Affiliated Hospital of Southwest Medical University, Luzhou, Sichuan, China; 4 Department of Ophthalmology, The First People’s Hospital of Yibin, Yibin, Sichuan, China

**Keywords:** inflammation, nutritional–metabolic status, post-stroke depression (PSD), serum prealbumin, spontaneous intracerebral hemorrhage (sICH)

## Abstract

**Background:**

Post-stroke depression (PSD) represents a frequent neuropsychiatric condition in patients with spontaneous intracerebral hemorrhage (sICH) and is associated with impaired functional rehabilitation and diminished quality of life. Clarifying biological factors associated with subsequent PSD may improve the understanding of psychological vulnerability after sICH. Serum prealbumin, a negative acute-phase protein that may reflect acute inflammatory and nutritional–metabolic status, has been associated with adverse stroke outcomes, but its relationship with PSD remains unclear. This study aimed to investigate the association between serum prealbumin at admission and PSD at 3 months post-stroke.

**Methods:**

A retrospective cohort study was conducted on 446 patients with sICH. Serum prealbumin at admission was defined as the first available measurement obtained within 48 hours after hospital admission. PSD was defined as a diagnosis of depression at 3 months post-stroke, based on the *Diagnostic and Statistical Manual of Mental Disorders, Fifth Edition* criteria and a 17-item Hamilton Depression Rating Scale (HAMD-17) score of ≥8. The association between serum prealbumin and PSD was assessed through multivariable logistic regression analysis.

**Results:**

In total, 159 patients (35.7%) were diagnosed with PSD. Serum prealbumin levels were reduced in patients with PSD compared with those without PSD (223.7 ± 52.8 vs. 243.4 ± 53.6 mg/L, *P* < 0.001). Patients in the third and fourth quartiles of serum prealbumin had significantly lower odds of PSD. Higher admission serum prealbumin was independently associated with lower odds of PSD after multivariable adjustment.

**Conclusions:**

Lower admission serum prealbumin was independently associated with higher odds of PSD at 3 months among patients with sICH who completed follow-up assessment. Serum prealbumin may represent a nonspecific peripheral marker reflecting acute inflammatory and nutritional–metabolic status after sICH.

## 1 Introduction

Post-stroke depression (PSD) frequently occurs in individuals recovering from stroke, with a prevalence of about one-third, and is associated with delayed rehabilitation, reduced quality of life, and heightened mortality risk (1). Although most studies on PSD have focused on the acute ischemic stroke (AIS) population, PSD is also common among patients with spontaneous intracerebral hemorrhage (sICH) (2, 3). In a recent comparative study, the reported prevalence of PSD was 42.3% in patients with sICH and 22.9% in those with AIS (4), underscoring the potential burden of mood disturbances in the hemorrhagic stroke population. However, evidence regarding the mechanisms, epidemiological characteristics, and intervention strategies related to PSD specifically in sICH patients remains limited (5). These patients are frequently underrepresented in existing research, often excluded from major clinical trials, leading to a substantial gap in evidence-based management for this subgroup. Identifying biological markers associated with subsequent PSD may improve our understanding of psychological vulnerability after sICH.

Importantly, ischemic stroke and sICH differ substantially in their underlying injury mechanisms and clinical trajectories. In sICH, hematoma-related mass effect, blood-product toxicity, perihematomal edema, blood–brain barrier disruption, and pronounced inflammatory activation may influence both circulating prealbumin concentrations and the subsequent development of depressive symptoms. In addition, sICH-specific characteristics, including hematoma location, intraventricular extension, and neurosurgical treatment, introduce biological and clinical heterogeneity that may be obscured when ischemic and hemorrhagic stroke populations are pooled. Therefore, evaluating this association in an sICH cohort may provide more disease-specific evidence and reduce heterogeneity related to stroke subtype.

Both nutritional status and systemic inflammation have been increasingly linked to the onset of neuropsychiatric disorders following stroke (6, 7). Serum albumin, a routinely measured negative acute-phase protein historically used in nutritional assessment, has been associated with depression and unfavorable stroke outcomes (8, 9). However, albumin has a relatively long circulating half-life of approximately 20 days and may respond slowly to acute changes. In contrast, prealbumin, also known as transthyretin (TTR), has a substantially shorter half-life of approximately 2 days and may therefore respond more rapidly to acute inflammatory activation and metabolic stress. Prealbumin is structurally and functionally distinct from albumin and participates in the transport of thyroxine and the retinol-binding protein–retinol complex. Following stroke, systemic inflammatory activation, reduced nutritional intake, and metabolic stress may suppress hepatic prealbumin synthesis and lower its circulating concentration. These disturbances have also been implicated in the pathophysiology of depression through neuroinflammatory, neuroendocrine, and neurotransmitter-related pathways. Therefore, low serum prealbumin may reflect an integrated state of inflammatory and metabolic vulnerability relevant to the subsequent development of PSD. Notably, experimental studies have reported that hippocampal TTR overexpression induces depression-like behavior, whereas TTR-null mice exhibit fewer signs of depression-like behavior (10, 11). This apparent discrepancy may reflect compartment- and context-specific effects of TTR. Circulating TTR is predominantly synthesized by the liver, whereas TTR in the cerebrospinal fluid is mainly produced by the choroid plexus. Therefore, findings from experimental hippocampal overexpression or lifelong genetic deletion cannot be directly equated with a single serum prealbumin measurement in patients with sICH. Lower serum prealbumin levels have been associated with higher mortality, poorer functional outcomes, and an increased risk of complications across a range of clinical conditions, including stroke, critical illness, cardiovascular disease, and cancer (12–17). A previous study reported an association between lower admission serum prealbumin and PSD at 1 month after acute stroke. However, stroke-subtype-specific analyses were not reported (18), and evidence regarding this association at later follow-up remains limited. The 3-month time point falls within the period when many PSD episodes first emerge and facilitates comparison with previous studies (19). Therefore, this study aimed to determine whether serum prealbumin at admission was associated with PSD at 3 months among patients with sICH.

## 2 Materials and methods

### 2.1 Research design and participants

This was a single-center, retrospective observational cohort study conducted in the Department of Neurosurgery at the Affiliated Hospital of Southwest Medical University, a Class A tertiary teaching hospital in Luzhou, Sichuan, China. Clinical data from patients with sICH who were hospitalized between January 2022 and December 2023 were retrospectively analyzed.

The study was approved by the Medical Ethics Committee of the Affiliated Hospital of Southwest Medical University (Approval No. [KY2025433]). Informed consent was waived, as the study involved retrospective analysis of previously recorded electronic medical records and did not include any disclosure of or interference with patient privacy. All study procedures were performed in compliance with the Declaration of Helsinki and applicable ethical guidelines.

Inclusion criteria were as follows:

1. Patients aged ≥18 years;
2. Patients with a diagnosis of sICH confirmed by cranial computed tomography (CT) within 24 hours of onset;
3. Patients who underwent serum prealbumin testing as part of routine clinical care within 48 hours of hospital admission.

Exclusion criteria were as follows:

1. Patients with traumatic intracerebral hemorrhage or secondary causes, such as brain tumor, ruptured aneurysm, or arteriovenous malformation;
2. Patients with severe hepatic or renal dysfunction;
3. Patients with a history of depression (clinical diagnosis or previous treatment) or other serious mental illnesses;
4. Patients with severe immune system diseases (e.g., systemic lupus erythematosus, rheumatoid arthritis), active infection, malignant tumors, or significant thyroid dysfunction;
5. Patients transferred from outside hospitals after surgery, as postoperative status may affect the interpretation of early laboratory measurements;
6. Patients who were unable to complete the 3-month follow-up after admission due to early death, severe impairment of consciousness, or aphasia.

Delirium was not a prespecified exclusion criterion and was not systematically assessed using a standardized instrument.

### 2.2 Clinical information collection

(1) Demographic and socioeconomic information: Demographic and socioeconomic characteristics collected at admission included age (categorized as <60, 60–74, or ≥75 years), sex (male or female), marital status (categorized as married or cohabiting, or single, divorced, or widowed), education level (categorized as less than high school, high school, or more than high school), occupation type (categorized as farmer, unemployed, retired, or employee), monthly income level (categorized as <¥1000, ¥1000–2999, ¥3000–4999, and ≥¥5000), and body mass index (BMI, kg/m²; categorized as <24, ≥24 to <28, or ≥28). Standardized measures of social support, family caregiving resources, loneliness, and psychological resilience were not available in the retrospective dataset.

(2) Health-related behaviors and vascular risk conditions: Behavioral data—such as smoking status and alcohol consumption—were collected at admission through patient or caregiver reports and recorded as binary variables (yes/no). Regular physical activity was defined as performing moderate-intensity exercise or labor on ≥3 days per week for ≥30 minutes per day for at least 3 months prior to the index sICH, and was recorded as a binary variable (yes/no) based on patient or caregiver report. Vascular risk conditions, including hypertension, diabetes mellitus, previous stroke, and heart disease, were obtained from self-reports or family-provided medical histories. Previous stroke was defined as any documented history of stroke before the index sICH event, based on medical records.

(3) Medical insurance type: Medical insurance type was categorized as either urban employee basic medical insurance (UEBMI) or non-UEBMI (including urban-rural resident insurance or having no insurance coverage).

(4) Location of sICH and treatment modality: According to admission imaging data, the location of sICH was categorized as left or right basal ganglia, left or right lobar regions, intraventricular hemorrhage (IVH), or infratentorial hemorrhage. Whether the patient underwent neurosurgical intervention was also recorded.

In this study, information from patients’ imaging reports was retrospectively extracted to document the bleeding site and morphology. Hematoma volume could not be uniformly obtained for the entire cohort because some imaging reports did not provide a numeric estimate, and the original CT images were not consistently retrievable for standardized measurement. Therefore, hematoma volume was not included in the primary analysis models. However, numeric hematoma volume was available from radiology reports for 383 patients (85.9%) and was incorporated in complete-case sensitivity analyses.

(5) Assessment of neurological function and functional status: Neurological function was evaluated by a trained neurologist in the initial 24-hour period following admission. Evaluation of stroke severity was performed with the 15-item National Institutes of Health Stroke Scale (NIHSS), which assesses parameters such as consciousness level, speech, ocular function, and limb strength. Higher total scores reflect more severe neurological impairment. Functional status was assessed using the modified Rankin Scale (mRS) and the Barthel Index (BI). The mRS evaluates the degree of disability (score range: 0–6), while the BI measures independence in 10 activities of daily living (score range: 0–100), with higher scores indicating better functional outcomes.

### 2.3 Laboratory parameters

Laboratory parameters were measured from peripheral venous blood samples collected within 48 hours after hospital admission. For the purposes of this study, “serum prealbumin at admission” was defined as the first available serum prealbumin measurement obtained within this period. The exact interval from stroke onset to blood collection and whether specific acute treatments had been initiated before sampling were not systematically recorded. Serum albumin was measured using the bromocresol green (BCG) colorimetric method, and serum prealbumin was determined by the transmission immunoturbidimetric method. Hemoglobin was assessed using the sodium lauryl sulfate (SLS) method on an automated hematology analyzer. All laboratory assessments were performed by the Clinical Laboratory Department, whose staff were blinded to participants’ clinical data. Quality control protocols were strictly followed, and all tests were performed using standardized automated equipment.

### 2.4 Assessment of depressive symptoms

Patients were followed up at 3 months post-stroke via telephone interview, video consultation, or outpatient visit; however, the specific modality was not consistently documented for each participant. Assessment of depressive symptoms was conducted with the 17-item Hamilton Depression Rating Scale (HAMD-17), administered by certified evaluators. The evaluators were blinded to the patients’ clinical and laboratory data. PSD was identified according to the *Diagnostic and Statistical Manual of Mental Disorders, Fifth Edition* (DSM-5) criteria, combined with a total score of ≥8 on the HAMD-17. This cutoff has been empirically supported in previous studies of post-stroke populations (20). Depression assessment was scheduled at approximately 3 months after the index sICH. This time point falls within the period when most PSD episodes first occur (19).

### 2.5 Statistical analysis

Continuous variables were assessed for normality within the non-PSD and PSD groups using the Shapiro–Wilk test, and homogeneity of variances was evaluated using Levene’s test. Normally distributed continuous variables with equal variances were summarized as mean ± standard deviation (SD) and compared using the independent-samples Student’s t-test. If the assumption of equal variances was violated, Welch’s t-test was used. Non-normally distributed continuous variables were summarized as median and interquartile range (IQR) and compared using the Mann–Whitney U test. Categorical variables were described as frequencies and proportions (n [%]) and compared using the Pearson’s chi-square test or Fisher’s exact test. BMI data were incomplete in 2 participants (0.45%). The missing anthropometric values were replaced with the corresponding overall median values before BMI was calculated. All other variables included in the primary multivariable models were complete. Admission BI score, serum albumin, and hemoglobin were missing in 2 (0.45%), 6 (1.35%), and 1 (0.22%) participants, respectively. Because these variables had low proportions of missingness, their summaries and between-group comparisons were based on available cases without imputation. Hematoma volume was unavailable in 63 participants (14.1%) and was addressed separately through a complete-case sensitivity analysis among 383 participants with available volume data.

To examine the association between serum prealbumin and PSD, unadjusted, partially adjusted, and fully adjusted logistic regression models were constructed. Multicollinearity among covariates was assessed before modeling based on the criteria of variance inflation factor (VIF) < 5 and tolerance > 0.1 (21). Due to collinearity between the BI score and the admission mRS score, only mRS was retained in the models. Glasgow Coma Scale (GCS) scores were not available as a consistently recorded and standardized variable in the structured electronic medical record extract used for the present analysis. Consequently, a reliable patient-level missingness rate could not be determined, and GCS was not included in the multivariable models. Admission NIHSS and mRS scores were included as available measures of neurological severity and functional status. Covariates were selected according to clinical relevance and previous evidence rather than univariable statistical significance or automated variable-selection procedures. The partially adjusted model controlled for available demographic and socioeconomic variables, including age, sex, marital status, BMI, occupation type, and medical insurance type. The fully adjusted model further incorporated clinical and behavioral variables: admission mRS score, admission NIHSS score, regular physical activity, alcohol consumption, history of diabetes mellitus, and previous stroke. Serum prealbumin was included in all models as the primary exposure of interest.

As an exploratory analysis, the associations of serum prealbumin and serum albumin with 3-month PSD were evaluated among participants with available serum albumin measurements. To ensure comparability, each biomarker was entered separately as a continuous variable into a fully adjusted logistic regression model using the same analytic population and the same covariates as Model 3. This analysis was intended to compare the observed association patterns rather than to establish the diagnostic, predictive, or clinical superiority of one biomarker over the other.

To further characterize the shape and robustness of the association, serum prealbumin was categorized into quartiles (Q1–Q4) based on its distribution in the study population. Multivariable logistic regression analyses were conducted using Q1 (<203 mg/L) as the reference group. A trend across quartiles was evaluated by modeling prealbumin quartiles as an ordinal variable (coded 1–4) in the multivariable logistic regression models (*P* for trend). This quartile-based trend analysis approach has been widely applied in observational studies investigating the association between inflammatory or nutritional biomarkers and depression, such as a recent study evaluating systemic immune–inflammatory index (SII) and depression in U.S. adults with hypertension (22). The quartile cutoff values were defined as: Q1 (<203 mg/L), Q2 (203–236 mg/L), Q3 (237–272 mg/L), and Q4 (>272 mg/L), with group sizes of 114, 112, 108, and 112 patients, respectively. The covariate adjustment strategies used in the quartile and trend analyses were consistent with those applied in the partially and fully adjusted models. To assess potential nonlinearity, restricted cubic spline analysis with four knots placed at the 5th, 35th, 65th, and 95th percentiles of the serum prealbumin distribution was performed within the fully adjusted logistic regression model. Nonlinearity was assessed using a likelihood-ratio test comparing the spline model with the corresponding model containing only a linear term for serum prealbumin.

Exploratory subgroup analyses were conducted by sex, age group, BMI category, medical insurance type, and neurosurgery status. Models were adjusted for age, sex, categorical admission mRS score, alcohol consumption, regular physical activity, and medical insurance type, except when the corresponding variable was examined as the subgroup factor. Effect modification was assessed using multiplicative interaction terms. Global interaction tests were used for age and BMI. No adjustment was made for multiple comparisons.

As a sensitivity analysis, we performed complete-case multivariable logistic regression adjusting for hematoma volume among patients with available volume data (n = 383). Hematoma volume was log-transformed (natural logarithm) to account for its right-skewed distribution. Additional sensitivity analyses modeled hematoma volume on its original continuous scale and as a dichotomous variable (≤30 vs. >30 mL).

Statistical analyses were performed using the Statistical Package for the Social Sciences (SPSS), version 29.0 (IBM Corp., Armonk, NY, USA), and GraphPad Prism 11 (GraphPad Software, Boston, MA, USA). Restricted cubic spline analyses were conducted using R software, version 4.4.1 (R Foundation for Statistical Computing, Vienna, Austria). All statistical tests were two-sided, and *P* < 0.05 was considered statistically significant.

## 3 Results

### 3.1 Comparison of baseline characteristics

**Figure 1:**
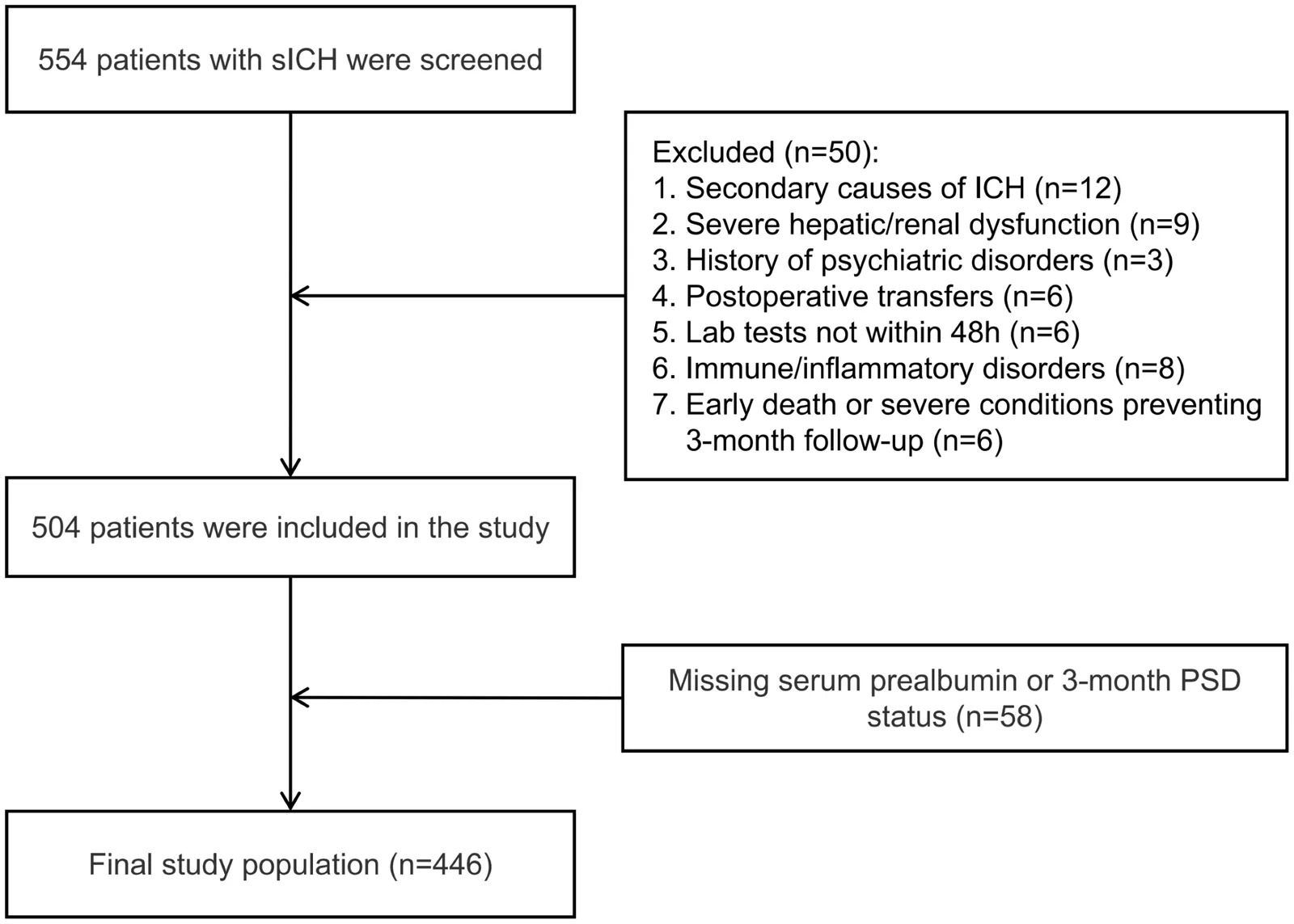
Flowchart of patient selection and exclusions. sICH, spontaneous intracerebral hemorrhage; PSD, post-stroke depression.

In total, 554 individuals with sICH were initially evaluated, of whom 504 met the initial clinical eligibility criteria. Of these, 58 were excluded due to missing serum prealbumin or 3-month PSD data, resulting in a final analytic sample of 446 patients (**Figure 1**). Among them, 159 patients (35.7%) were diagnosed with PSD at 3 months.

**Figure 2:**
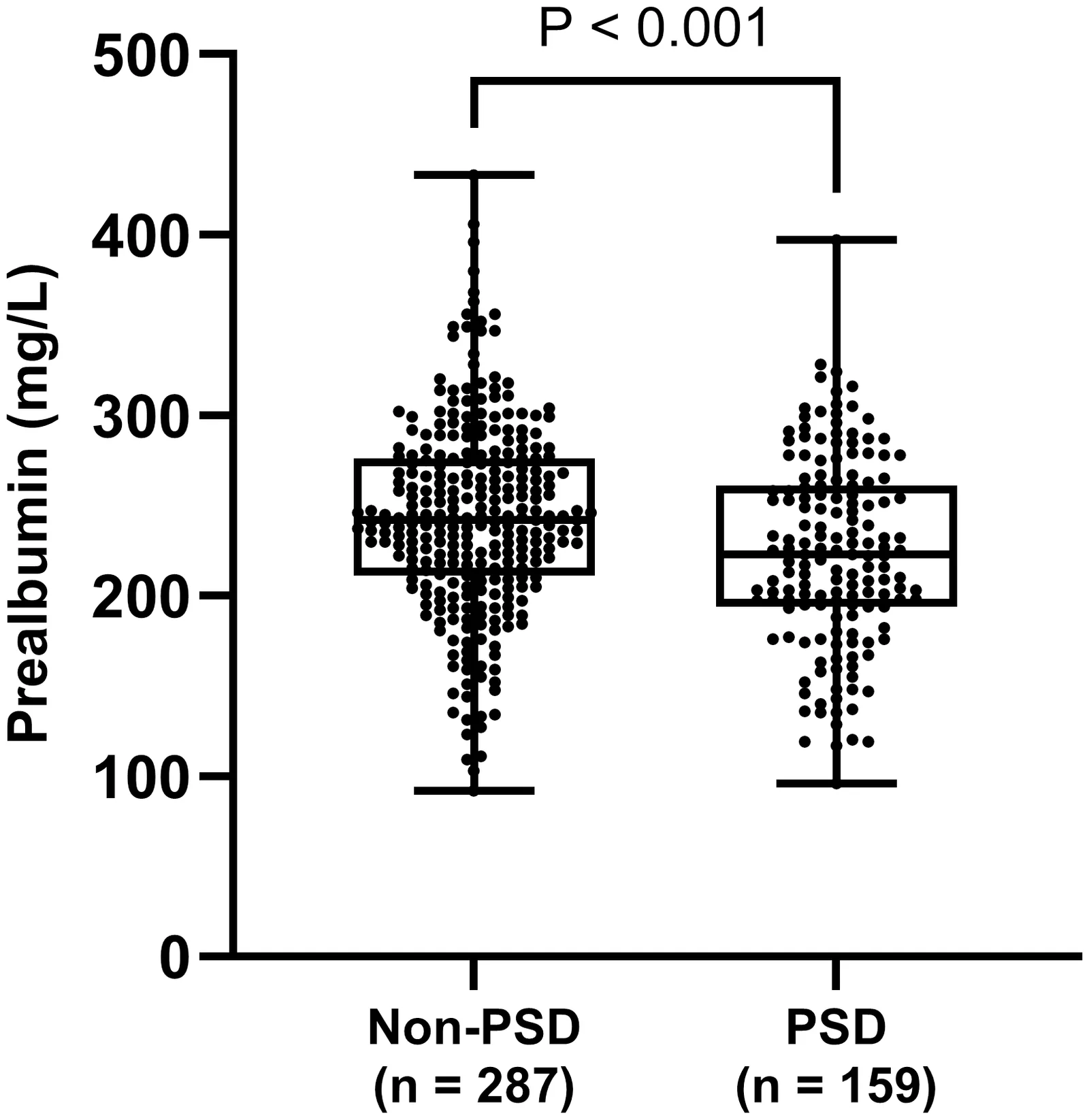
Distribution of admission serum prealbumin levels according to 3-month post-stroke depression status. Individual dots represent individual patients. Boxes indicate the interquartile range (25th–75th percentiles), horizontal lines within the boxes indicate the median, and whiskers indicate the minimum and maximum values. Admission serum prealbumin levels were significantly lower in patients with PSD than in those without PSD (independent-samples Student’s t-test, *P* < 0.001). PSD, post-stroke depression.

As shown in **Table 1**, compared with the non-PSD group, patients in the PSD group were more likely to be unemployed or engaged in farming (*P* = 0.028), had a significantly lower rate of UEBMI coverage (18.2% vs. 33.1%, *P* < 0.001), and were less likely to consume alcohol (20.1% vs. 31.0%, *P* = 0.013) or have diabetes mellitus (5.0% vs. 12.2%, *P* = 0.014). Moreover, a smaller proportion of patients in the PSD group engaged in regular physical activity (21.4% vs. 30.3%, *P* = 0.042). At admission, patients in the PSD group exhibited greater neurological impairment and functional disability, as reflected by lower BI scores and higher mRS and NIHSS scores (all *P* < 0.001). Serum prealbumin was also significantly lower among patients with PSD (223.7 ± 52.8 vs. 243.4 ± 53.6 mg/L, *P* < 0.001), suggesting a potential association between compromised nutritional or inflammatory status at admission and the subsequent development of PSD. The distribution of admission serum prealbumin levels in the non-PSD and PSD groups is further illustrated in **Figure 2**.

**Table 1:** Baseline profiles of PSD and non-PSD patients.

Characteristics	Overall (n = 446)	Non-PSD (n = 287)	PSD (n = 159)	*P*
Age				0.117
<60 years	233 (52.2%)	153 (53.3%)	80 (50.3%)	
60–74 years	164 (36.8%)	109 (38.0%)	55 (34.6%)	
≥75 years	49 (11.0%)	25 (8.7%)	24 (15.1%)	
Sex				0.141
Male	319 (71.5%)	212 (73.9%)	107 (67.3%)	
Female	127 (28.5%)	75 (26.1%)	52 (32.7%)	
Marital status				0.152
Married or cohabiting	407 (91.3%)	266 (92.7%)	141 (88.7%)	
Single, divorced, or widowed	39 (8.7%)	21 (7.3%)	18 (11.3%)	
BMI (kg/m²)				0.146
<24	137 (30.7%)	81 (28.2%)	56 (35.2%)	
≥24 to <28	263 (59.0%)	179 (62.4%)	84 (52.8%)	
≥28	46 (10.3%)	27 (9.4%)	19 (11.9%)	
Education level				0.079
Less than high school	247 (55.4%)	153 (53.3%)	94 (59.1%)	
High school	155 (34.8%)	99 (34.5%)	56 (35.2%)	
More than high school	44 (9.9%)	35 (12.2%)	9 (5.7%)	
Occupation type				**0.028**
Farmer	200 (44.8%)	117 (40.8%)	83 (52.2%)	
Unemployed	68 (15.2%)	41 (14.3%)	27 (17.0%)	
Retired	55 (12.3%)	42 (14.6%)	13 (8.2%)	
Employee	123 (27.6%)	87 (30.3%)	36 (22.6%)	
Monthly income level				0.089
<¥1000	149 (33.4%)	95 (33.1%)	54 (34.0%)	
¥1000–2999	102 (22.9%)	67 (23.3%)	35 (22.0%)	
¥3000–4999	99 (22.2%)	55 (19.2%)	44 (27.7%)	
≥¥5000	96 (21.5%)	70 (24.4%)	26 (16.4%)	
Smoking				0.711
No	298 (66.8%)	190 (66.2%)	108 (67.9%)	
Yes	148 (33.2%)	97 (33.8%)	51 (32.1%)	
Alcohol consumption				**0.013**
No	325 (72.9%)	198 (69.0%)	127 (79.9%)	
Yes	121 (27.1%)	89 (31.0%)	32 (20.1%)	
Regular physical activity				**0.042**
No	325 (72.9%)	200 (69.7%)	125 (78.6%)	
Yes	121 (27.1%)	87 (30.3%)	34 (21.4%)	
Previous stroke				0.718
No	385 (86.3%)	249 (86.8%)	136 (85.5%)	
Yes	61 (13.7%)	38 (13.2%)	23 (14.5%)	
Hypertension				0.230
No	113 (25.3%)	78 (27.2%)	35 (22.0%)	
Yes	333 (74.7%)	209 (72.8%)	124 (78.0%)	
Diabetes mellitus				**0.014**
No	403 (90.4%)	252 (87.8%)	151 (95.0%)	
Yes	43 (9.6%)	35 (12.2%)	8 (5.0%)	
History of heart disease				0.695
No	421 (94.4%)	270 (94.1%)	151 (95.0%)	
Yes	25 (5.6%)	17 (5.9%)	8 (5.0%)	
Medical insurance type				**<0.001**
Non-UEBMI	322 (72.2%)	192 (66.9%)	130 (81.8%)	
UEBMI	124 (27.8%)	95 (33.1%)	29 (18.2%)	
Neurosurgery				0.100
No	358 (80.3%)	237 (82.6%)	121 (76.1%)	
Yes	88 (19.7%)	50 (17.4%)	38 (23.9%)	
Location of sICH				0.067
Left basal ganglia	126 (28.3%)	79 (27.5%)	47 (29.6%)	
Right basal ganglia	120 (26.9%)	66 (23.0%)	54 (34.0%)	
Left lobar region	38 (8.5%)	28 (9.8%)	10 (6.3%)	
Right lobar region	28 (6.3%)	21 (7.3%)	7 (4.4%)	
IVH	85 (19.1%)	56 (19.5%)	29 (18.2%)	
Infratentorial	49 (11.0%)	37 (12.9%)	12 (7.5%)	
Neurological and functional status				
Admission BI score	45.0 [25.0, 80.0]	60.0 [35.0, 90.0]	30.0 [20.0, 60.0]	**<0.001**
Admission mRS score	3.0 [2.0, 4.0]	3.0 [2.0, 4.0]	4.0 [3.0, 5.0]	**<0.001**
Admission NIHSS score	8.0 [3.0, 15.0]	6.0 [2.0, 12.0]	10.0 [6.0, 18.0]	**<0.001**
Laboratory parameters				
Albumin (g/L)	42.5 [39.8, 44.7]	42.6 [40.1, 44.8]	42.1 [39.4, 44.2]	0.137
Prealbumin (mg/L)	236.4 ± 54.1	243.4 ± 53.6	223.7 ± 52.8	**<0.001**
Hemoglobin (g/L)	137.0 [126.0, 148.0]	137.5 [128.0, 148.0]	136.0 [123.0, 148.0]	0.226
Hematoma volume (mL)	9.0 [4.0, 19.0]	8.0 [3.0, 19.0]	11.0 [5.0, 20.0]	0.082

Data are presented as n (%), mean ± standard deviation (SD), or median [interquartile range, IQR], as appropriate. *P* values were calculated using Pearson’s chi-square test or Fisher’s exact test for categorical variables and Student’s t-test, Welch’s t-test, or Mann–Whitney U test for continuous variables, according to the distributional characteristics of the data. Bold text values represent *P* values less than 0.05. Two participants had incomplete anthropometric data required for BMI calculation; the missing values were replaced with the corresponding overall medians before BMI was calculated. Admission BI score, serum albumin, hemoglobin, and hematoma volume were analyzed using available cases without imputation; the corresponding sample sizes were 444, 440, 445, and 383, respectively. After median substitution for the incomplete anthropometric data, all variables required for the primary multivariable logistic regression models were available, and all 446 participants were included in the primary regression analyses. Regular physical activity was defined as moderate-intensity physical activity or labor performed for ≥30 min/day on ≥3 days/week for at least 3 months prior to stroke. PSD, post-stroke depression; BMI, body mass index; IQR, interquartile range; SD, standard deviation; mRS, modified Rankin Scale; NIHSS, National Institutes of Health Stroke Scale; BI, Barthel Index; sICH, spontaneous intracerebral hemorrhage; IVH, intraventricular hemorrhage; UEBMI, Urban Employee Basic Medical Insurance.

### 3.2 Association between serum prealbumin and PSD

**Table 2** shows that higher serum prealbumin was associated with lower odds of PSD across all three logistic regression models. In the fully adjusted quartile analysis, participants in Q3 and Q4 had lower odds of PSD than those in Q1, although the point estimates were not strictly monotonic (Q3: OR = 0.408, 95% CI: 0.212–0.785; Q4: OR = 0.476, 95% CI: 0.243–0.932). A *post hoc* contrast showed no statistically significant difference between Q4 and Q3 (*P* = 0.644). An overall inverse trend was observed across quartiles (*P* for trend = 0.017), and restricted cubic spline analysis showed no statistically significant evidence of departure from linearity (*P* for nonlinearity = 0.713).

**Table 2:** Association between serum prealbumin and 3-month PSD.

	Model 1		Model 2		Model 3	
	OR (95% CI)	*P*	OR (95% CI)	*P*	OR (95% CI)	*P*
**Prealbumin**	0.993 (0.989–0.997)	**0.001**	0.994 (0.989–0.998)	**0.003**	0.995 (0.990–0.999)	**0.023**
Prealbumin quartile						
Q1	**Reference**		**Reference**		**Reference**	
Q2	0.556 (0.326–0.947)	**0.031**	0.541 (0.306–0.955)	**0.034**	0.579 (0.312–1.073)	0.083
Q3	0.367 (0.209–0.644)	**<0.001**	0.378 (0.206–0.695)	**0.002**	0.408 (0.212–0.785)	**0.007**
Q4	0.418 (0.242–0.722)	**0.002**	0.451 (0.242–0.840)	**0.012**	0.476 (0.243–0.932)	**0.030**
***P* for trend**		**<0.001**		**0.006**		**0.017**

Model 1 is unadjusted. Model 2 is adjusted for age, sex, marital status, BMI, occupation type, and medical insurance type. Model 3 is additionally adjusted for admission mRS score, admission NIHSS score, regular physical activity, alcohol consumption, history of diabetes mellitus, and previous stroke. Bold text values represent *P* values less than 0.05. PSD, post-stroke depression; OR, odds ratio; CI, confidence interval; BMI, body mass index; mRS, modified Rankin Scale; NIHSS, National Institutes of Health Stroke Scale.

Among the covariates included in the fully adjusted model, reported alcohol consumption (adjusted OR = 0.548, 95% CI: 0.312–0.962, *P* = 0.036) and a history of diabetes mellitus (adjusted OR = 0.366, 95% CI: 0.149–0.898, *P* = 0.028) were also inversely associated with PSD.

### 3.3 Exploratory comparison of serum prealbumin and albumin

Among the 440 participants with available serum albumin measurements, higher serum prealbumin remained inversely associated with 3-month PSD in the fully adjusted model (OR per 1-mg/L increase = 0.9951, 95% CI: 0.9906–0.9996, *P* = 0.035). In contrast, serum albumin was not significantly associated with PSD in the same analytic population using identical covariate adjustment (OR per 1-g/L increase = 0.9672, 95% CI: 0.9162–1.0210, *P* = 0.227; **Table 3**). These results represent a comparison of association patterns and do not establish a statistically significant difference or clinical superiority between the two biomarkers.

**Table 3:** Exploratory comparison of the associations of serum prealbumin and serum albumin with 3-month PSD among participants with available serum albumin measurements (n = 440).

Biomarker	Unit of increase	Adjusted OR (95% CI)	*P*
Serum prealbumin	Per 1 mg/L	0.9951 (0.9906–0.9996)	**0.035**
Serum albumin	Per 1 g/L	0.9672 (0.9162–1.0210)	0.227

Each biomarker was entered separately into a fully adjusted logistic regression model based on the same 440 participants and adjusted for the same covariates as Model 3 in **Table 2**. Bold text values represent *P* values less than 0.05. PSD, post-stroke depression; OR, odds ratio; CI, confidence interval.

### 3.4 Subgroup analyses and assessment of interaction effects

Exploratory subgroup analyses showed no statistically significant evidence of effect modification by sex (*P* for interaction = 0.571), age group (*P* = 0.217), BMI category (*P* = 0.238), medical insurance type (*P* = 0.077), or neurosurgery status (*P* = 0.580; **Supplementary Table 1**). These findings should be interpreted cautiously because several strata contained limited numbers of participants and PSD events, and no adjustment was made for multiple comparisons.

### 3.5 Sensitivity analyses

A complete-case sensitivity analysis excluding the 2 participants with incomplete BMI data yielded materially unchanged results (adjusted OR = 0.994, 95% CI: 0.990–0.999, *P* = 0.013; n = 444). Hematoma volume data were unavailable for 63 of 446 participants (14.1%). Patients with missing hematoma volume were more likely to have markers of greater hemorrhage severity and intensive management, including higher rates of neurosurgery and intraventricular hemorrhage, whereas admission prealbumin levels and the prevalence of 3-month PSD were comparable between participants with and without hematoma volume data (**Supplementary Table 2**). In the hematoma-volume-available subcohort (n = 383), the inverse association remained after additional adjustment for natural-log-transformed hematoma volume (adjusted OR = 0.994, 95% CI: 0.989–0.999, *P* = 0.021; **Supplementary Table 3**). The results remained materially unchanged when hematoma volume was modeled on its original continuous scale (adjusted OR = 0.994, *P* = 0.019) or dichotomized at 30 mL (adjusted OR = 0.994, *P* = 0.016).

## 4 Discussion

Most previous studies of PSD did not distinguish between stroke types or focused mainly on patients with ischemic stroke (23). By restricting the analysis to patients with sICH, the present study reduced heterogeneity related to stroke subtype and provided evidence specific to the studied hemorrhagic stroke population. Among the included survivors of sICH, the prevalence of PSD at 3 months was 35.7%, comparable to the high burden reported in prior sICH cohorts during follow-up (2, 3). In this retrospective study, the 3-month follow-up point was used because depression assessments at this stage were most consistently available in the dataset. This time frame also lies within the period when most PSD episodes first occur (19), facilitating comparability across studies. Assessing depressive symptoms beyond the acute hospitalization period may help reduce the influence of immediate post-event stressors and acute neurological fluctuations. Higher admission serum prealbumin was associated with lower odds of PSD at 3 months, and this association persisted after multivariable adjustment. Quartile analyses showed that participants in Q3 and Q4 had lower odds of PSD than those in Q1, with an overall inverse trend across quartiles. Although the quartile-specific point estimates were not strictly monotonic, the difference between Q3 and Q4 was not statistically significant. Restricted cubic spline analysis also showed no statistically significant evidence of nonlinearity. These findings support an overall inverse association but do not establish a strictly monotonic dose–response relationship; a threshold or plateau pattern cannot be completely excluded. No statistically significant evidence of interaction was observed across the examined subgroups; however, the limited sample sizes in some strata may have reduced the power to detect heterogeneity.

In the exploratory analysis restricted to participants with available serum albumin measurements, serum prealbumin remained inversely associated with PSD after multivariable adjustment, whereas serum albumin was not significantly associated with PSD in the same analytic population. This pattern may be consistent with the shorter circulating half-life of prealbumin and its potentially greater responsiveness to acute inflammatory and nutritional–metabolic changes after sICH. However, a statistically significant association for prealbumin and a nonsignificant association for albumin do not, by themselves, demonstrate a statistically significant difference between the two biomarkers. Because the present study did not conduct a formal head-to-head biomarker-performance analysis and was not designed for prediction-model development, these findings do not establish the diagnostic, predictive, or general clinical superiority of serum prealbumin over serum albumin.

Several biological pathways may help explain the observed inverse association between serum prealbumin and PSD. Following sICH, blood-product toxicity, blood–brain barrier disruption, perihematomal inflammation, and systemic inflammatory activation may be accompanied by increased concentrations of inflammatory mediators, including C-reactive protein (CRP), tumor necrosis factor α (TNF-α), and interleukin-6 (IL-6) (24, 25). Inflammatory processes have also been associated with major depressive disorder and PSD through neuroinflammatory, neuroendocrine, and neurotransmitter-related pathways (26, 27). As a negative acute-phase protein, serum prealbumin may decrease in response to systemic inflammatory activation, and previous studies have reported inverse correlations between serum prealbumin and inflammatory markers such as CRP and IL-6 in other clinical settings (28–30). Therefore, lower serum prealbumin in the present study may reflect greater acute inflammatory burden rather than a depression-specific causal process.

Lower serum prealbumin may also reflect reduced nutritional intake, acute catabolic stress, and altered metabolic demands after sICH. Malnutrition has been associated with depressive symptoms and unfavorable recovery in patients with stroke and other chronic diseases (6, 31, 32), and nutritional disturbances have been linked to neuroplasticity, oxidative stress, gut microbiota, and mitochondrial function (33). Thus, serum prealbumin may represent a nonspecific peripheral correlate of combined inflammatory and nutritional–metabolic vulnerability after sICH. However, because the present study did not assess inflammatory biomarkers, nutritional intake, longitudinal changes in prealbumin, or formal mediation pathways, it cannot determine whether serum prealbumin directly participates in the development of PSD.

Given its short circulating half-life, admission serum prealbumin should be interpreted as a cross-sectional peripheral correlate of acute inflammatory and nutritional–metabolic status rather than as a stable individual characteristic. Although lower early serum prealbumin was associated with PSD assessed at 3 months, this finding does not indicate that prealbumin remained low throughout follow-up or that the early reduction exerted a persistent biological effect. Because prealbumin was measured only once, we could not determine whether low levels were transient or persistent, whether subsequent normalization was associated with the occurrence of PSD, or whether longitudinal changes in prealbumin were related to the course of depressive symptoms. Accordingly, the present data cannot determine whether serial monitoring would provide clinically actionable information or guide the timing, frequency, or duration of psychological, nutritional, or other interventions. Prospective studies incorporating repeated prealbumin measurements are needed to clarify the clinical relevance of prealbumin trajectories.

Prior research from our group has highlighted the association between elevated inflammatory cytokines and depression- and anxiety-related symptoms in patients with glioma, based on multiplex cytokine profiling. These findings underscore the contribution of systemic inflammation to affective disturbances in neurological disorders (34). Building on this framework, the present study evaluated serum prealbumin alongside clinical, behavioral, and socioeconomic factors to examine whether its association with PSD remained after multivariable adjustment.

It is worth noting that the significant association between serum prealbumin and PSD is not isolated. This study further found that patients who developed PSD were more likely to exhibit both socioeconomic and clinical disadvantages at baseline. The PSD group included a greater proportion of unemployed individuals and those covered by non-UEBMI plans—such as urban–rural resident insurance or no insurance at all—compared with the non-PSD group. This is consistent with previous studies, suggesting that socioeconomic disadvantage may increase susceptibility to PSD (35). In line with this, other studies have reported that financial burden and lack of social support can amplify the psychological stress associated with stroke, thereby increasing the risk of depression (36, 37). In addition, the lower rate of regular physical activity observed in the PSD group suggests that lifestyle factors may influence mood regulation through inflammatory or neurometabolic pathways (38, 39). In terms of neurological and functional status, patients with PSD had significantly lower admission BI scores and higher admission mRS and NIHSS scores, further supporting the notion that the severity of post-stroke disability is strongly associated with depressive symptoms (4, 40). Both reported alcohol consumption and a history of diabetes mellitus were inversely associated with PSD in the fully adjusted model. These counterintuitive associations should be interpreted cautiously and should not be considered evidence that alcohol consumption or diabetes mellitus protects against PSD. Although some population-based studies have reported inverse associations between light-to-moderate alcohol consumption and depression (41, 42), alcohol exposure in the present study was recorded only as a binary yes/no variable. Information on drinking frequency, quantity, duration, beverage type, and previous drinking status was unavailable. We were therefore unable to determine whether participants reporting alcohol consumption were light, moderate, or heavy drinkers, or to distinguish lifetime abstainers from former drinkers. The observed association may consequently have been influenced by exposure misclassification, underreporting, and residual confounding related to socioeconomic, behavioral, or premorbid health factors. Given the complex and potentially bidirectional relationship between alcohol use and depression (43, 44), our findings do not support alcohol consumption as a preventive or therapeutic strategy for PSD. The inverse association between diabetes mellitus and PSD should likewise be regarded as exploratory. Only 43 participants had a history of diabetes mellitus, of whom 8 met the study-defined criteria for PSD, resulting in a limited number of exposed outcome events and a potentially unstable estimate. Furthermore, diabetes mellitus was recorded only as a binary medical-history variable, without information on disease duration, glycemic control, diabetic complications, or antidiabetic treatment. Residual confounding, incomplete characterization of diabetes severity, selection of survivors who were able to complete the 3-month depression assessment, and chance may have contributed to the unexpected direction of this association. Therefore, neither finding should be interpreted causally, and both require confirmation in larger prospective cohorts with more detailed exposure assessment.

The present findings indicate that higher admission serum prealbumin was independently associated with lower odds of PSD at 3 months after adjustment for relevant demographic, clinical, behavioral, and socioeconomic factors. However, serum prealbumin should not be interpreted as a standalone screening, diagnostic, or risk-stratification tool for PSD, and its incremental value as part of a multivariable screening model has not been established. Reduced serum prealbumin may instead represent a nonspecific marker of greater inflammatory and nutritional–metabolic vulnerability during the acute phase of sICH. This interpretation is consistent with the multifactorial nature of PSD, which involves biological disturbances, neurological severity and disability, behavioral factors, and psychosocial circumstances (26, 40, 45). Prospective multicenter studies incorporating serial prealbumin measurements, inflammatory biomarkers, and detailed nutritional and psychosocial assessments are required to replicate this association in independent cohorts and determine its clinical relevance.

This study has several limitations that warrant consideration. First, as a single-center retrospective analysis, the findings may be subject to selection and information bias, and residual confounding cannot be fully excluded despite multivariable adjustment. In addition, GCS was not consistently available in the structured dataset, and residual confounding related to level of consciousness cannot be excluded. Delirium was not systematically assessed using a standardized instrument; therefore, particularly hypoactive delirium may have been underrecognized. Because delirium may coexist with acute medical complications and nutritional impairment, residual confounding related to delirium cannot be excluded. Information on the timing of in-hospital complications, including infections, seizures, and electrolyte disturbances, relative to blood sampling was not consistently available, limiting our ability to evaluate their potential influence on serum prealbumin levels and subsequent depressive symptoms. In addition, depressive symptoms at 3 months were assessed via outpatient visits, video consultations, or telephone interviews, and the specific modality was not systematically recorded for each participant; thus, we could not formally test inter-modality agreement or adjust for follow-up modality, which may have introduced measurement heterogeneity in HAMD-17 scoring. Second, hematoma volume was missing for a subset of participants and may not be completely at random; however, sensitivity analyses yielded materially similar conclusions (**Supplementary Tables 2, 3**). Third, serum prealbumin was measured only once within 48 hours of admission, which limited our ability to capture dynamic changes in inflammatory and nutritional status. The exact interval from stroke onset to blood collection, treatments administered before sampling, and nutritional intake before blood collection were not systematically recorded. Therefore, reduced food intake during the early post-stroke period may have contributed to lower serum prealbumin levels, and the measured concentration should be interpreted as an early in-hospital value rather than a strictly pretreatment baseline measurement. Fourth, although several socioeconomic indicators were available, direct measures of social support, family caregiving resources, loneliness, and psychological resilience were not collected, which may have resulted in residual confounding. Fifth, key inflammatory markers such as CRP and IL-6 were not included in the models due to their limited availability and inconsistent measurement in the retrospective dataset. Finally, the analysis focused solely on depressive status at a single time point (3 months), which may not fully reflect the long-term trajectory of psychological outcomes following stroke. Future research should adopt a multicenter prospective design incorporating serial biomarker assessments and multidimensional psychosocial measures to clarify the temporal relationship between serum prealbumin and PSD.

## 5 Conclusions

In this single-center retrospective cohort of patients with sICH who completed 3-month follow-up assessment, lower admission serum prealbumin was independently associated with higher odds of PSD. The inverse association remained after multivariable adjustment and in the sensitivity analysis additionally accounting for hematoma volume. Serum prealbumin may reflect acute inflammatory and nutritional–metabolic vulnerability; however, the observational design and single measurement preclude causal or clinical-utility inferences. Prospective multicenter studies incorporating serial biomarker measurements are needed to confirm this association and clarify its biological significance.

## Data Availability

The datasets for this article are not publicly available because they contain sensitive patient information and are therefore subject to ethical and privacy restrictions. De-identified data may be obtained from the corresponding author upon reasonable request and with appropriate institutional approvals. Requests to access these datasets should be directed to Jie Zhou, Email zj000718@yeah.net.
