# Supplementary material for: Serum prealbumin at admission is associated with post-stroke depression at 3 months in patients with spontaneous intracerebral hemorrhage
Source: Front Psychiatry. 2026 Sep 10;17:1698928. doi: 10.3389/fpsyt.2026.1698928 (PMC13601338; doi:10.3389/fpsyt.2026.1698928)
Supplement: Supplementary file 1 [file Table1.docx]

**Supplementary Table 1. Exploratory subgroup analyses of the association between serum prealbumin and 3-month PSD.** ORs and 95% CIs are reported per 1-mg/L increase in serum prealbumin. Models were adjusted for age, sex, categorical admission mRS score, alcohol consumption, regular physical activity, and medical insurance type, except when used as the subgroup variable. *P* for interaction was derived from multiplicative interaction terms; global tests were used for age and BMI. No adjustment was made for multiple comparisons. Bold text values represent *P* values less than 0.05. Abbreviations: PSD, post-stroke depression; OR, odds ratio; CI, confidence interval; BMI, body mass index; mRS, modified Rankin Scale; UEBMI, Urban Employee Basic Medical Insurance.

| **Variable** | **Subgroup** | **OR (95% CI)** | ***P*** | ***P* for interaction** |
| --- | --- | --- | --- | --- |
|  |  |  |  |  |
| **Sex** |  |  |  | 0.571 |
|  | Male | 0.994  (0.990–0.999) | **0.029** |  |
|  | Female | 0.997  (0.988–1.007) | 0.592 |  |
| **Age** |  |  | | 0.217 |
|  | <60 years | 0.995  (0.989–1.001) | 0.075 |  |
|  | 60–74 years | 0.998  (0.991–1.005) | 0.577 |  |
|  | ≥75 years | 0.979  (0.959–0.999) | **0.040** |  |
| **BMI (kg/m²)** |  |  |  | 0.238 |
|  | <24 | 0.991  (0.984–0.998) | **0.012** |  |
|  | ≥24 to <28 | 0.998  (0.992–1.004) | 0.539 |  |
|  | ≥28 | 0.992  (0.976–1.008) | 0.322 |  |
| **Medical insurance type** |  |  | | 0.077 |
|  | Non-UEBMI | 0.997  (0.992–1.002) | 0.231 |  |
|  | UEBMI | 0.988  (0.978–0.997) | **0.011** |  |
| **Neurosurgery** |  |  |  | 0.580 |
|  | No | 0.995  (0.990–1.000) | **0.032** |  |
|  | Yes | 0.997  (0.989–1.006) | 0.540 |  |
