# Supplementary material for: Serum prealbumin at admission is associated with post-stroke depression at 3 months in patients with spontaneous intracerebral hemorrhage
Source: Front Psychiatry. 2026 Sep 10;17:1698928. doi: 10.3389/fpsyt.2026.1698928 (PMC13601338; doi:10.3389/fpsyt.2026.1698928)
Supplement: Supplementary file 2 [file Table2.docx]

**Supplementary Table 2. Baseline characteristics by hematoma volume availability.** Data are presented as n (%), mean ± standard deviation (SD), or median [interquartile range, IQR], as appropriate. *P* values were calculated using Pearson’s chi-square test or Fisher’s exact test for categorical variables and Student’s t-test, Welch’s t-test, or Mann–Whitney U test for continuous variables, according to the distributional characteristics of the data. Bold text values represent *P* values less than 0.05. Abbreviations: PSD, post-stroke depression; BMI, body mass index; IQR, interquartile range; SD, standard deviation; mRS, modified Rankin Scale; NIHSS, National Institutes of Health Stroke Scale; sICH, spontaneous intracerebral hemorrhage; IVH, intraventricular hemorrhage.

| **Characteristics** | **Overall**  **(n = 446)** | **Volume available**  **(n = 383)** | **Volume missing**  **(n = 63)** | ***P*** |
| --- | --- | --- | --- | --- |
| **Age** |  |  |  | 0.565 |
| <60 years | 233 (52.2%) | 204 (53.3%) | 29 (46.0%) |  |
| 60–74 years | 164 (36.8%) | 138 (36.0%) | 26 (41.3%) |  |
| ≥75 years | 49 (11.0%) | 41 (10.7%) | 8 (12.7%) |  |
| **Sex** |  |  |  | 0.749 |
| Male | 319 (71.5%) | 275 (71.8%) | 44 (69.8%) |  |
| Female | 127 (28.5%) | 108 (28.2%) | 19 (30.2%) |  |
| **BMI (kg/m²)** |  |  |  | 0.463 |
| <24 | 137 (30.7%) | 115 (30.0%) | 22 (34.9%) |  |
| ≥24 to <28 | 263 (59.0%) | 226 (59.0%) | 37 (58.7%) |  |
| ≥28 | 46 (10.3%) | 42 (11.0%) | 4 (6.3%) |  |
| **Neurosurgery** |  |  |  | **<0.001** |
| No | 358 (80.3%) | 320 (83.6%) | 38 (60.3%) |  |
| Yes | 88 (19.7%) | 63 (16.4%) | 25 (39.7%) |  |
| **Location of sICH** |  |  |  | **0.005** |
| Left basal ganglia | 126 (28.3%) | 116 (30.3%) | 10 (15.9%) |  |
| Right basal ganglia | 120 (26.9%) | 105 (27.4%) | 15 (23.8%) |  |
| Left lobar region | 38 (8.5%) | 34 (8.9%) | 4 (6.3%) |  |
| Right lobar region | 28 (6.3%) | 23 (6.0%) | 5 (7.9%) |  |
| IVH | 85 (19.1%) | 62 (16.2%) | 23 (36.5%) |  |
| Infratentorial | 49 (11.0%) | 43 (11.2%) | 6 (9.5%) |  |
| **Admission mRS score** | 3.0  [2.0, 4.0] | 3.0  [2.0, 4.0] | 3.0  [2.0, 4.5] | 0.914 |
| **Admission NIHSS score** | 8.0  [3.0, 15.0] | 8.0  [3.0, 14.0] | 8.0  [2.5, 17.0] | 0.860 |
| **Prealbumin (mg/L)** | 236.4 ± 54.1 | 236.0 ± 52.2 | 238.5 ± 64.6 | 0.775 |
| **PSD** |  |  |  | 0.679 |
| No | 287 (64.3%) | 245 (64.0%) | 42 (66.7%) |  |
| Yes | 159 (35.7%) | 138 (36.0%) | 21 (33.3%) |  |
