# Supplementary material for: Serum prealbumin at admission is associated with post-stroke depression at 3 months in patients with spontaneous intracerebral hemorrhage
Source: Front Psychiatry. 2026 Sep 10;17:1698928. doi: 10.3389/fpsyt.2026.1698928 (PMC13601338; doi:10.3389/fpsyt.2026.1698928)
Supplement: Supplementary file 3 [file Table3.docx]

**Supplementary Table 3. Multivariable logistic regression analysis of factors associated with PSD in the hematoma-volume-available subcohort (n = 383).** Model 1 is unadjusted. Model 2 is adjusted for age, sex, marital status, BMI, occupation type, and medical insurance type. Model 3 is additionally adjusted for admission mRS score, admission NIHSS score, regular physical activity, alcohol consumption, history of diabetes mellitus, ln-transformed hematoma volume, and previous stroke. Bold text values represent *P* values less than 0.05. Abbreviations: PSD, post-stroke depression; OR, odds ratio; CI, confidence interval; BMI, body mass index; mRS, modified Rankin Scale; NIHSS, National Institutes of Health Stroke Scale.

|  | **Model 1** | | **Model 2** | | **Model 3** | |
| --- | --- | --- | --- | --- | --- | --- |
|  | **OR (95% CI)** | ***P*** | **OR (95% CI)** | ***P*** | **OR (95% CI)** | ***P*** |
| **Prealbumin** | 0.993  (0.988–0.997) | **<0.001** | 0.993  (0.988–0.998) | **0.005** | 0.994  (0.989–0.999) | **0.021** |
| **Prealbumin quartile** |  |  |  |  |  |  |
| Q1 | **Reference** |  | **Reference** |  | **Reference** |  |
| Q2 | 0.594  (0.338–1.043) | 0.070 | 0.536  (0.292–0.983) | **0.044** | 0.580  (0.301–1.118) | 0.104 |
| Q3 | 0.359  (0.193–0.668) | **0.001** | 0.359  (0.184–0.702) | **0.003** | 0.378  (0.186–0.770) | **0.007** |
| Q4 | 0.412  (0.228–0.744) | **0.003** | 0.429  (0.217–0.845) | **0.014** | 0.453  (0.219–0.936) | **0.033** |
| ***P* for trend** |  | **<0.001** |  | **0.007** |  | **0.017** |
